# Disturbed skin barrier in children with chronic kidney disease

**DOI:** 10.1007/s00467-014-2932-2

**Published:** 2014-08-17

**Authors:** Elzbieta Wojtowicz-Prus, Katarzyna Kilis-Pstrusinska, Adam Reich, Katarzyna Zachwieja, Monika Miklaszewska, Maria Szczepanska, Jacek C. Szepietowski

**Affiliations:** 1 Department of Dermatology, Regional Specialist Hospital, Center for Research and Development, Wroclaw, Poland; 2 Department of Paediatric Nephrology, Wroclaw Medical University, Borowska 213, 50-556 Wroclaw, Poland; 3 Department of Dermatology, Venereology and Allergology, Wroclaw Medical University, Wroclaw, Poland; 4 Department of Paediatric Nephrology, Polish-American Children’s Hospital, Jagiellonian University, Krakow, Poland; 5 Clinic of Paediatrics, Nephrology and Endocrinology, Silesian Medical University, Zabrze, Poland

**Keywords:** Dry skin, Chronic kidney disease, Dialysis, Conservative treatment, Children

## Abstract

**Background:**

There are limited data on skin lesions in children with end-stage renal failure. The aim of the study was an evaluation of the skin barrier in children with different stages of chronic kidney disease (CKD). The prevalence of xerosis, its severity, as well as its link selected demographic factors, were examined.

**Methods:**

The study included 103 children: 72 with CKD stages 3–5 (38 on conservative treatment and 34 on dialysis) and 31 patients with primary monosymptomatic nocturnal enuresis as a control group. Initially, the study subjects described the localisation and severity of dry skin by themselves. Next, clinical evaluation of xerosis, non-invasive corneometric assessment of epidermis moisturising and the measurement of transepidermal water loss were performed.

**Results:**

Most CKD children reported dry skin. The problem of xerosis was identified more frequently in patients on dialysis (67.6 %) than on conservative treatment (42.1 %) (*p* = 0.01). CKD patients divided according to skin dryness did not differ with regards to age, sex, initial kidney disease and CKD duration.

**Conclusions:**

Disturbed skin barrier is an important concern of children with CKD, intensifying as the disease progresses. This symptom occurs on early stages of CKD and it should be taken into consideration in the CKD management.

## Introduction

Chronic kidney disease (CKD), regardless of its cause, may be accompanied by various skin lesions. The most common symptom in adults suffering from CKD is pruritus. Other signs include xerosis, skin hyperpigmentation, ecchymoses, acquired perforating dermatoses, nail lesions, calcinosis cutis, porphyria cutanea tarda, as well as eczematous lesions at the site of an arteriovenous fistula and skin infections [1–3].

Dry skin is a common problem in patients with CKD. Thomas et al. [1] reported its presence in 66.7 % of adult patients with CKD. Other authors showed similar results [2, 4, 5]. Histopathological examination of the skin of CKD patients reveals microangiopathy, atrophy of the epidermis, sebaceous glands, secretory tubules and ducts of the eccrine glands, hyperkeratosis and elastin fibre fragmentation [6–8]. Diminished sweat secretion lowers skin hydration and impairs elimination of electrolytes, urea, amino acids, proteins and lipids [9, 10]. The level of glycerol—one of the relevant endogenous humectants and a component of a natural moisturising factor—is also decreased. Xerosis may be observed in all skin areas; however, in some patients it is more severe on the lower legs [8]. Skin dryness may appear at various stages of CKD, but it is more frequently diagnosed in dialysis subjects (45 %) [11]. The data regarding the clinical relevance of skin dryness in patients with CKD are very limited. However, it seems probable that the disturbed skin barrier observed in xerosis may facilitate the development of skin infections.

There are very limited data on skin lesions in children with CKD. Only a few papers concerning xerosis in paediatric patients have been published and they present the results of studies on a small number of patients who needed renal replacement therapy [2, 11, 12]. An assessment of the skin barrier in children with CKD in the earlier stages of the disease could not be found in the contemporary literature; therefore, we conducted a study with the aim of evaluating skin dryness in children with different stages of CKD. The prevalence of xerosis, its severity, as well as its link with selected demographic factors characteristic of CKD, were examined.

## Materials and methods

### Patients

The study involved 103 children from three Polish paediatric nephrology centres located in Wroclaw, Krakow and Zabrze. It included 72 patients with CKD at stages 3–5 [13] and 31 patients with primary monosymptomatic nocturnal enuresis as a control group. Thirty-eight patients with CKD were given conservative treatment and 34 were provided with renal replacement therapy (20 haemodialysis subjects and 14 peritoneal dialysis subjects). All dialysis patients were combined into one group, as the numbers of patients who were subjected to haemodialysis and peritoneal dialysis were low.

Children with CKD had not been on any dermatological treatment, nor had any of them suffered from any infection or other condition (including atopic dermatitis), which might significantly influence the skin hydration, for at least 4 weeks before enrolment into our study. Haemodialysis was performed three times a week for 4 h using polysulphone dialysers. Patients underwent dialysis using acetate and bicarbonate concentrate with standard potassium and calcium concentrations. All assessments were conducted during hospitalisation on days when haemodialysis was not performed. In the group of patients treated with peritoneal dialysis an automated peritoneal dialysis (APD) method was used. Baxter Peritoneal Dialysis Solutions (1.36 % glucose) were administered. Physioneal® and/or Extranil® were used if required. APD lasted 12 h on average. The examination was carried out during the patient’s stay in the nephrology centre.

None of children in the control group suffered from any chronic disease, except for primary monosymptomatic nocturnal enuresis. The children did not receive any pharmacotherapy, nor did they suffer from any acute infection or other acute condition within the period of 4 weeks before the study.

Before entering the study, children and their caretakers gave informed consent to participation in the study; they were also informed of their right to leave the study at any time. The study protocol was approved by the Bioethics Committee at the Wroclaw Medical University (opinion KB-751/2012).

### Study design

The skin barrier in children was assessed using several methods. Initially, all study subjects were asked to describe the location and severity of skin xerosis using the terms absent, mild, moderate or severe dryness. If necessary, the severity of skin xerosis was reported with the help of parents (especially in very young children). Next, a clinical evaluation of skin dryness, a non-invasive corneometric assessment of epidermis moisturising and a measurement of transepidermal water loss (TEWL) using a tewameter were performed. All measurements were performed on four areas of the skin: the forearm, the lower leg, the abdomen and the chest under stable environmental conditions: temperature 20–22 °C and air humidity 40–50 % after a 10-min rest in the sitting position.

#### Evaluation of xerosis

Clinical evaluation of xerosis was conducted using the standard clinical four-point Xerosis Assessment Scale (0—normal skin, without any xerosis; 1—mild xerosis; 2—moderately dry skin with minimal flaking; 3—severe xerosis, heavy scaling visible). This scale has been used in a number of previous studies on xerosis [14, 15].

#### Corneometric assessment of epidermis moisture

The assessment of epidermis moisturising was performed using a Corneometer CM825 produced by Courage + Khazaka Electronic (Cologne, Germany). This measurement is based on the different dielectric constants of water and other substances. The measuring capacitor shows changes in capacitance according to the moisture content of the samples. An electric scatter field penetrates the skin during the measurement and the dielectricity is determined. Results achieved using the Corneometer are demonstrated in arbitrary units (AU). A lowering of these values indicates a decrease in the water content in the outer layers of the epidermis.

#### Assessment of transepidermal water loss

Transepidermal water loss was performed using a Tewameter TM300 produced by Courage + Khazaka Electronic. A certain evaporation of water from the skin always takes place as part of the normal skin metabolism. As soon as the barrier function of the skin alters, the water loss increases. Therefore, this measurement remains the basis for much cosmetic and dermatological research. The Tewameter® probe measures the density gradient of the water evaporation from the skin by two pairs of sensors (temperature and relative humidity) inside the hollow cylinder. A microprocessor analyses the gradient of these values and after calculation expresses the evaporation rate in g/h/m^2^.

### Statistical analysis

Contingencies and frequencies (percentage values) were calculated for qualitative parameters, while means and standard deviations for normally distributed quantitative variables or medians and quartiles for skewed variables were measured. All data were processed using Microsoft Office Excel 2010 software (Microsoft Corporation, Warsaw, Poland) and Statistica 10.0 (StatSoft, Krakow, Poland). Chi-squared test, Student’s *t* test, the Mann–Whitney *U* test, analysis of variance and Spearman rank correlation test were used, where appropriate. The statistical significance level was set at 0.05.

## Results

The demographic data of the groups studied are presented in Table 1. The most common cause of CKD was congenital malformation of the urinary tract, which was present in 36 patients (50 %): 23 of them (32 %) were on conservative treatment and 13 (18 %) were on dialysis. Other patients had CKD resulting from polycystic kidney disease—13 children (18 %; 7 on conservative treatment and 6 on dialysis), chronic glomerulonephritis—10 children (14 %; 3 on conservative treatment and 7 on dialysis), acute tubulointerstitial nephritis—8 children (11 %; 4 on conservative treatment and 4 on dialysis). The remaining 5 children (7 %) had Alport syndrome, tubulopathy or other, unknown causes—1 patient underwent conservative treatment and 4 had dialysis.

**Table 1:** Characteristics of the examined groups. Continuous data given as means ± SD (without range)

	Control group *n* = 31	CKD on conservative treatment *n* = 38	CKD on dialysis *N* = 34	*p*
Age (years)	10.7 ± 3.9	11.0 ± 4.5	11.1 ± 4.2	0.95
Gender: female/male (%)	17/14 (54.8/45.2)	8/30 (21.1/78.9)	22/12 (64.7/35.3)	<0.001
Cause of CKD				
Anomaly of the urinary tract	–	23 (60.5)	13 (38.2)	0.2
Polycystic kidney disease	–	7 (18.4)	6 (17.6)	
Chronic glomerulonephritis	–	3 (7.9)	7 (20.6)	
Chronic interstitial nephropathy	–	4 (10.5)	4 (11.8)	
Other	–	1 (2.6)	4 (11.8)	
CKD duration (years)	–	7.3 ± 4.9	7.4 ± 4.8	0.98
CKD stage				
3	–	20	0	
4	–	18	0	
5	–	0	34	

*CKD* chronic kidney disease

Table 2 shows the results of a questionnaire on the prevalence, severity and site of skin dryness in the study subjects. The vast majority of children in the control group (81.1 %) did not report any skin dryness. This problem, however, was frequently reported by the CKD patients, more often in those undergoing dialysis (67.6 % of them) than in the patients receiving conservative treatment (42.1 %; overall, 54.2 % patients with CKD had dry skin symptoms). The differences between the control group and the study group, as well as between the two groups of patients with CKD, were statistically significant (*p* < 0.001 and *p* = 0.03 respectively). Children from the control group reported skin dryness exclusively on the forearm and lower legs; the patients with CKD additionally reported this symptom on the abdomen and the chest. Detailed data are provided in Table 2.

**Table 2:** Prevalence, severity and site of skin dryness and pruritus in the study subjects

	Control group, *n* (%)	CKD patients on conservative treatment, *n* (%)	CKD patients on dialysis, *n* (%)	*p*
Skin dryness					
None	27 (81.1)	22 (57.9)	11 (32.4)	<0.001
Mild	4 (12.9)	15 (39.5)	17 (50.0)	
Moderate	0 (0)	1 (2.6)	6 (17.6)	
Prevalence of dry skin					
Forearm	3 (9.7)	3 (7.9)	8 (23.5)	0.12
Lower leg	3 (9.7)	9 (23.7)	12 (35.3)	0.05
Abdomen	0 (0)	3 (7.7)	5 (14.7)	0.09
Chest	0 (0)	1 (2.6)	4 (11.8)	0.06
Presence of pruritus	0 (0)	7 (18.4)	8 (23.5)	0.01

*CKD* chronic kidney disease

In the control group the median duration of skin dryness was 2 years (25 –75 %: 1–15, range: 1–15), and in CKD patients undergoing conservative treatment and dialysis it was 6 years (25–75 %: 3–8, range: 0.5–15) and 8 years (25–75 %: 3.7–14, range: 1.5–17) respectively. The differences observed were not statistically significant (*p* = 0.31). The subgroups of CKD patients created based on the presence of skin dryness did not differ with regard to age (*p* = 0.3), sex (*p* = 0.77), initial kidney disease (*p* = 0.43) and the duration of CKD (*p* = 0.43). A statistically significant difference was observed for the severity of skin dryness in the abdominal area and lower legs between the CKD and the control group. No significant differences were noted with regard to the clinical evaluation of skin dryness at other sites (Table 3).

**Table 3:** Assessment of dry skin (results demonstrated as mean ± SD)

	Control group	CKD patients on conservative treatment	CKD patients on dialysis	*p*
Clinical evaluation of dry skin				
Forearm	0.1 ± 0.3	0.13 ± 0.41	0.24 ± 0.43	0.35
Lower leg	0.1± 0.3	0.3 ± 0.5	0.4 ± 0.5	0.03
Abdomen	0	0.1 ± 0.3	0.2 ± 0.5	0.04
Thorax	0	0.03 ± 0.2	0.1 ± 0.3	0.13
Epidermis moisturising (corneometry; AU)				
Forearm	31.2 ± 6.2	30.4 ± 0.9.8	28.4 ± 5.7	0.32
Lower leg	31.8 ± 7.7	25.9 ± 7.2	24.5 ± 6.6	<0.001
Abdomen	35.6 ± 7.8	29.5 ± 0.10.1	25.1 ± 7.0	<0.001
Thorax	45.5 ± 7.8	40.4 ± 13.5	33.8 ± 7.7	<0.001
Transepidermal water loss (g/m^2^/h)				
Forearm	5.5 ± 2.9	8.5 ± 4.1	8.3 ± 3.1	<0.001
Lower leg	6.7 ± 3.6	7.9 ± 5.0	9.8 ± 5.3	0.04
Abdomen	6.9 ± 5.3	9.6 ± 6.4	9.2 ± 5.5	0.12
Thorax	6.7 ± 3.7	9.5 ± 6.8	7.6 ± 3.3	0.06

*CKD* chronic kidney disease

There were statistically significant differences between the groups regarding epidermis moisturising on the forearm, lower leg, abdomen and chest. A significant difference was also noted in the thoracic area for patients on conservative treatment and undergoing dialysis, as well as in the forearm and lower leg between the control group and other groups (as well as between CKD patients treated with different methods) with regard to TEWL (Table 3). Correlations between the subjective assessment of dry skin and TEWL and corneometry for all children are demonstrated in Table 4.

**Table 4:** Correlations between the subjective assessment of skin dryness and corneometry and transepidermal water loss (TEWL) for all subjects (*n* = 103)

	Clinically assessed skin dryness			
	Forearm	Lower leg	Abdomen	Chest
Epidermis moisturising (corneometry)	R =−0.38, *p* < 0.001	R = −0.52, *p* < 0.001	R = 0.33, *p* < 0.001	R = −0.32, *p* = 0.001
TEWL	R = 0.09, *p* = 0.38	R = 0.13, *p* = 0.2	R = 0.13, *p* = 0.2	R = 0.18, *p* = 0.07

*TEWL* transepidermal water loss

## Discussion

In this study concerning the paediatric population, skin dryness was considerably more frequently reported by children with CKD (54.2 %) than by children from the control group (18.9 %). Children with CKD undergoing dialysis reported skin dryness more often than those receiving conservative treatment. Similar data were described by other authors in adults with CKD. Anderson et al. [16] estimated that 50–70 % of adults on dialysis suffer from xerosis. In the study by Thomas et al. [1] the total prevalence of skin dryness in adults with different stages of CKD was 66.7 %, and in most cases affected haemodialysis patients. In the recently published study by Kolla et al. [17], including the group of 100 patients undergoing haemodialysis, aged 49 ± 12.3 years, skin dryness was the most common cutaneous problem, and was observed in 78.3 % subjects.

A slightly lower prevalence of dry skin in our study compared with data of other authors may be associated with the severity of CKD and the patient’s age. To the best of our knowledge this is the first study to evaluate skin moisture in children with CKD undergoing conservative treatment. Studies on adults with CKD mainly included patients on maintenance dialysis [5, 11, 18, 19]. In addition, cutaneous lesions may develop with aging of the skin [20]. Xerosis may also be influenced by such factors as race, accompanying illnesses and various environmental factors [21]. Nowadays, various devices allowing the evaluation of epidermis moisturising and TEWL are available, as were also used in our study. These methods of skin barrier assessment allowed us to standardise the subjective data obtained from the subjects studied.

In our study, skin dryness was more frequently reported on forearms and lower legs than at other sites. Similar results regarding the localisation of xerosis in adults undergoing haemodialysis were reported by Udayakumar et al. [2] and by Kato et al. [22]. However, some other authors stated that skin dryness in CKD patients may be present anywhere. Morton et al. [5] observed skin dryness in adults undergoing haemodialysis and peritoneal dialysis in all the areas examined with the greatest severity on the back. In their study the differences between haemodialysis and peritoneal dialysis subjects with regard to xerosis severity were noticeable, with more severe dryness in the first group. Results of corneometric examination were comparable with those of clinical xerosis assessments. Patients undergoing peritoneal dialysis had less skin moisture than patients undergoing haemodialysis and healthy controls in all the areas studied. Moreover, a significant correlation between lower moisturising of the *stratum corneum* and itching was found in both groups [5].

A complex analysis based on various methods leads us to the conclusion that more children suffering from CKD had skin dryness than healthy children and that it was most severe in patients on dialysis. It is very likely, that, similar to adult subjects [23], uremic xerosis may cause a significant decrease in the quality of life in children, although relevant data are lacking. Because skin dryness in CKD patients on dialysis was much more prevalent, more severe and covered larger areas than in other types of xerosis, in the past it was given the unique name *uraemic xerosis* [8]. Moreover, many studies suggested that uraemic xerosis is an important factor for uraemic pruritus [24–27]. Dry skin may also be a problem in healthy people. It is estimated that it affects about 15–20 % of general population [28]. However, unlike “physiological” dry skin, uraemic xerosis is typically associated with abnormalities in deeper layers of the epidermis (such as atrophy of sweat and sebaceous glands and their impaired secretory function), which may lead to decreased epidermis moisturising [8]. One of the possible causes of dry skin in dialysis patients may be abnormal pH in the *stratum corneum* [8, 25]. Elias et al. [29] pointed to pH level as one of the possible factors regulating the natural process of skin exfoliation in patients with ichthyosis. Decreased pH on the skin surface may activate various proteases, which play a role in *stratum corneum* exfoliation. It was observed that the pH of the *stratum corneum* is increased in dialysis patients [20], which may interrupt protease activation and, consequently, cause skin barrier dysfunction and xerosis in patients undergoing dialysis [8]. Remarkably, in adults undergoing haemodialysis, the use of emollients significantly reduced the dry skin and raised the quality of life of patients [23, 30]. In our opinion, emollients (applied at least two to three times a day) with/without oil baths are also the mainstay of the treatment of skin dryness in children with CKD. If necessary, keratolytics may be added, especially in older children.

There are some limitations of our study. The number of children with CKD is not large, but it seems to be representative. It has to be stressed that the population of children suffering from CKD is considerably smaller than the population of adult CKD patients. It is difficult to conduct such a study on a higher number of paediatric patients, since it is crucial to maintain the same conditions of the examinations and ensure that the examinations are performed by the same qualified dermatologist in order to provide uniform assessment criteria. In the future the difference between haemodialysis and peritoneal dialysis patients should be studied, as described for adult patients. Furthermore, the differences observed in 1 unit of TEWL between patients on dialysis and those on conservative treatment may be clinically meaningless, even though they are statistically significant.

In conclusion, dry skin seems to be a relevant problem in children with CKD. Xerosis was more prevalent in patients with CKD than in the reference group and in dialysed patients compared with patients undergoing conservative treatment, which indicates that it is aggravated with the progression of kidney function impairment. However, skin xerosis may also occur in the early stages of CKD and it should be taken into consideration when planning treatment in order to prevent skin lesion progression and improve the quality of life of children with CKD.
